# 3β-Chloro­cholest-5-en-7-one

**DOI:** 10.1107/S1600536810006380

**Published:** 2010-02-27

**Authors:** Mohd. Shaheen Khan, Othman Sulaiman, Rokiah Hashim, Madhukar Hemamalini, Hoong-Kun Fun

**Affiliations:** a School of Industrial Technology, Universiti Sains Malaysia, 11800 USM, Penang, Malaysia; b X-ray Crystallography Unit, School of Physics, Universiti Sains Malaysia, 11800 USM, Penang, Malaysia

## Abstract

The title compound, C_27_H_43_ClO, is a steroid derivative composed of a saturated carbon fused-ring framework with an alkyl side chain. The *A* and *C* rings have chair conformations and the *B* and *D* rings assume half-chair conformations. The cholesterol side chain is fully extended with a *gauche*, *trans* conformation of the terminal methyl groups. In the crystal structure, the molecules are aligned in an antiparallel fashion, forming alternate layers. These layers are then linked *via* C—H⋯O hydrogen bonds, forming a three-dimensional network.

## Related literature

For related structures, see: Kang *et al.* (1985 ▶); Yun *et al.* (1989 ▶); Ahn & Park (1990 ▶); Park & Shin (2002 ▶); Park (2004 ▶); Park *et al.* (2005 ▶). For the role of cholesterol derivatives in biological systems, see: Abrahamsson *et al.* (1977 ▶). For ring conformations, see: Cremer & Pople (1975 ▶). For the stability of the temperature controller used in the data collection, see: Cosier & Glazer (1986 ▶).
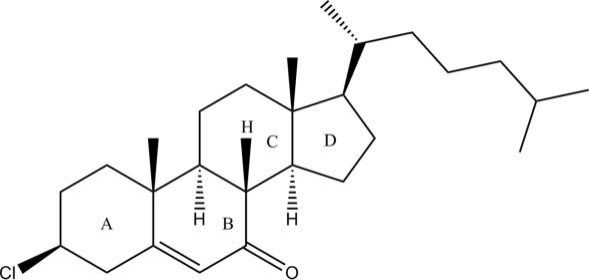


## Experimental

### 

#### Crystal data

C_27_H_43_ClO
*M* *_r_* = 419.06
Monoclinic, 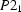

*a* = 11.1494 (11) Å
*b* = 7.8552 (8) Å
*c* = 14.6317 (14) Å
β = 109.535 (2)°
*V* = 1207.7 (2) Å^3^
*Z* = 2
Mo *K*α radiation
μ = 0.17 mm^−1^
*T* = 100 K
0.77 × 0.15 × 0.15 mm

#### Data collection

Bruker APEX DUO CCD area-detector diffractometer
Absorption correction: multi-scan (*SADABS*; Bruker, 2009 ▶) *T*_min_ = 0.878, *T*_max_ = 0.975
13366 measured reflections
6524 independent reflections
5728 reflections with *I* > 2s(*I*)
*R*_int_ = 0.027

#### Refinement

*R*[*F* ^2^ > 2σ(*F* ^2^)] = 0.038
*wR*(*F* ^2^) = 0.097
*S* = 1.06
6524 reflections
267 parameters
1 restraint
H-atom parameters constrained
Δρ_max_ = 0.35 e Å^−3^
Δρ_min_ = −0.23 e Å^−3^
Absolute structure: Flack (1983 ▶), 2745 Friedel pairs
Flack parameter: 0.00 (5)

### 

Data collection: *APEX2* (Bruker, 2009 ▶); cell refinement: *SAINT* (Bruker, 2009 ▶); data reduction: *SAINT*; program(s) used to solve structure: *SHELXTL* (Sheldrick, 2008 ▶); program(s) used to refine structure: *SHELXTL*; molecular graphics: *SHELXTL*; software used to prepare material for publication: *SHELXTL* and *PLATON* (Spek, 2009 ▶).

## Supplementary Material

Crystal structure: contains datablocks global, I. DOI: 10.1107/S1600536810006380/cv2698sup1.cif

Structure factors: contains datablocks I. DOI: 10.1107/S1600536810006380/cv2698Isup2.hkl

Additional supplementary materials:  crystallographic information; 3D view; checkCIF report

## Figures and Tables

**Table 1:** Hydrogen-bond geometry (Å, °)

*D*—H⋯*A*	*D*—H	H⋯*A*	*D*⋯*A*	*D*—H⋯*A*
C11—H11*B*⋯O1^i^	0.97	2.45	3.377 (2)	159
C14—H14*B*⋯O1^ii^	0.97	2.49	3.268 (2)	137

Symmetry codes: (i) 
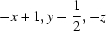
; (ii) 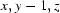.

**Fig. 1.**
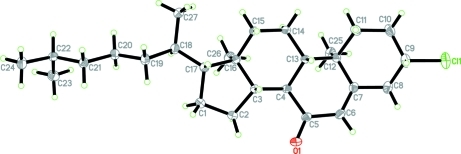
The molecular structure of (I) showing the atomic numbering and 30% probability displacement ellipsoids.

## supplementary crystallographic information

### Comment

Although the basic biological functions of cholesterol have been known for many years, little is known about its molecular interactions with enzymes, cholesterol-binding proteins and related effectors of gene transcription. A series of crystal structures of the esters and carbonates of cholesterol (Ahn & Park, 1990; Kang *et al.*, 1985; Yun *et al.*, 1989; Park & Shin, 2002; Park, 2004) has been examined in order to obtain structural information relevant to the liquid crystalline phases and the possible modes of association of the cholesterol derivatives themselves, as well as of other substances in biological systems (Abrahamsson *et al.*, 1977).  In view of the biological importance of cholesterols, we report here the crystal structure of the title compound (I) - a new cholesterol derivative.

In the asymmetric unit of the title compound (Fig. 1), the A (C7–C12) [Q = 0.5302 (18)Å, Θ = 168.67 (19)° and φ = 324.7 (10)°] and C (C13–C16/C3/C4) [0.5636 (17)Å, Θ = 172.25 (16)° and φ = 119.9 (13)°] rings have chair conformations (Cremer & Pople, 1975), and the B (C4–C7/C12–C13) [Q = 0.4830 (16)Å , Θ = 51 (19)° and φ =313.6 (2)°] and D (C1–C3/C16/C17) [Q = 0.4653 (17)Å and φ = 273.6 (2)°] rings assume half-chair conformations. The torsion angles  C20-C21-C22-C23 of 61.84 (18)° and C20-C21-C22-C24 of -175.12 (13)° show that the terminal isopropyl group has a (-)-gauche conformation. This type of conformation was also observed in the crystal structure of cholesteryl isobutylcarbonate (Park *et al.*, 2005).  There are eight chiral centres in the molecule. The absolute configurations of these sites were determined by the refinement of the Flack parameter. From the structure presented, these sites exhibit the following chiralities: C3 = S, C4 = S, C9 = S, C12 = R, C13 = S, C16 = R, C17 = R and C18 = R.

In the crystal structure, the molecules are aligned in an antiparallel fashion to form alternate layers. These layers are then linked via C—H···O (Table 1) hydrogen bonds to form a three dimensional network

### Experimental

A solution of butyl chromate [t-butyl alcohol (60 mL), CrO_3_ (20 g), acetic acid (35 mL) and acetic anhydride (10 mL)] was added at 0°C  to a solution of 3β-chlorocholest -5-ene (8 g) in CCl_4_ (150 mL), acetic acid (30 mL) and acetic anhydride (10 mL). The content was refluxed for 3 h and then diluted with water. The organic layer was washed with sodium bicarbonate solution (5%) and water and then dried over anhydrous sodium sulphate. Evaporation of the solvents under reduced pressure furnished 3β-chlorocholest-5-en-7-one which was crystallized from methanol (3.4 g), m. p. 144°C (reported, m. p.144 -145°C ).

### Refinement

All hydrogen atoms were positioned geometrically [C–H = 0.93–0.98Å] and were refined using a riding model, with *U*_iso_(H) = 1.2-1.5 *U*_eq_(C). 2745 Friedel pairs were used to determine the absolute configuration.

### Crystal data

**Table:**

C_27_H_43_ClO	*F*(000) = 460
*M**_r_* = 419.06	*D*_x_ = 1.152 Mg m^−^^3^
Monoclinic, *P*2_1_	Mo *K*α radiation, λ = 0.71073 Å
Hall symbol: P 2yb	Cell parameters from 5352 reflections
*a* = 11.1494 (11) Å	θ = 2.8–30.1°
*b* = 7.8552 (8) Å	µ = 0.17 mm^−^^1^
*c* = 14.6317 (14) Å	*T* = 100 K
β = 109.535 (2)°	Needle, pink
*V* = 1207.7 (2) Å^3^	0.77 × 0.15 × 0.15 mm
*Z* = 2	

### Data collection

**Table:**

Bruker APEX DUO CCD area-detector diffractometer	6524 independent reflections
Radiation source: fine-focus sealed tube	5728 reflections with *I* > 2s(*I*)
graphite	*R*_int_ = 0.027
φ and ω scans	θ_max_ = 30.1°, θ_min_ = 1.5°
Absorption correction: multi-scan (*SADABS*; Bruker, 2009)	*h* = −15→15
*T*_min_ = 0.878, *T*_max_ = 0.975	*k* = −11→11
13366 measured reflections	*l* = −20→20

### Refinement

**Table:**

Refinement on *F*^2^	Secondary atom site location: difference Fourier map
Least-squares matrix: full	Hydrogen site location: inferred from neighbouring sites
*R*[*F*^2^ > 2σ(*F*^2^)] = 0.038	H-atom parameters constrained
*wR*(*F*^2^) = 0.097	*w* = 1/[σ^2^(*F*_o_^2^) + (0.0473*P*)^2^ + 0.1133*P*] where *P* = (*F*_o_^2^ + 2*F*_c_^2^)/3
*S* = 1.06	(Δ/σ)_max_ = 0.001
6524 reflections	Δρ_max_ = 0.35 e Å^−^^3^
267 parameters	Δρ_min_ = −0.23 e Å^−^^3^
1 restraint	Absolute structure: Flack (1983); 2745 Friedel pairs
Primary atom site location: structure-invariant direct methods	Flack parameter: 0.00 (5)

### Special details

**Table:**

Experimental. The crystal was placed in the cold stream of an Oxford Cryosystems Cobra open-flow nitrogen cryostat (Cosier & Glazer, 1986) operating at 100.0 (1) K.
Geometry. All esds (except the esd in the dihedral angle between two l.s. planes) are estimated using the full covariance matrix. The cell esds are taken into account individually in the estimation of esds in distances, angles and torsion angles; correlations between esds in cell parameters are only used when they are defined by crystal symmetry. An approximate (isotropic) treatment of cell esds is used for estimating esds involving l.s. planes.
Refinement. Refinement of F^2^ against ALL reflections. The weighted R-factor wR and goodness of fit S are based on F^2^, conventional R-factors R are based on F, with F set to zero for negative F^2^. The threshold expression of F^2^ > 2sigma(F^2^) is used only for calculating R-factors(gt) etc. and is not relevant to the choice of reflections for refinement. R-factors based on F^2^ are statistically about twice as large as those based on F, and R- factors based on ALL data will be even larger.

### Fractional atomic coordinates and isotropic or equivalent isotropic displacement parameters (Å^2^)

**Table:**

	*x*	*y*	*z*	*U*_iso_*/*U*_eq_	
Cl1	0.93405 (4)	1.01064 (7)	−0.14116 (3)	0.03660 (12)	
O1	0.54188 (11)	1.39479 (15)	0.12534 (8)	0.0239 (2)	
C1	0.36679 (16)	1.1549 (2)	0.32152 (12)	0.0243 (3)	
H1A	0.2823	1.2007	0.2894	0.029*	
H1B	0.3900	1.1762	0.3906	0.029*	
C2	0.46410 (15)	1.2397 (2)	0.28114 (11)	0.0220 (3)	
H2A	0.4270	1.3372	0.2409	0.026*	
H2B	0.5394	1.2762	0.3333	0.026*	
C3	0.49592 (14)	1.09800 (19)	0.22116 (10)	0.0163 (3)	
H3A	0.4228	1.0886	0.1612	0.020*	
C4	0.61428 (14)	1.11592 (19)	0.19055 (10)	0.0153 (3)	
H4A	0.6889	1.1267	0.2493	0.018*	
C5	0.60913 (14)	1.2714 (2)	0.12715 (10)	0.0175 (3)	
C6	0.69051 (14)	1.2685 (2)	0.06594 (10)	0.0206 (3)	
H6A	0.6965	1.3672	0.0326	0.025*	
C7	0.75656 (14)	1.1313 (2)	0.05551 (10)	0.0185 (3)	
C8	0.84283 (15)	1.1441 (2)	−0.00546 (12)	0.0250 (3)	
H8A	0.9309	1.1469	0.0368	0.030*	
H8B	0.8251	1.2493	−0.0423	0.030*	
C9	0.82322 (14)	0.9942 (3)	−0.07473 (10)	0.0259 (3)	
H9A	0.7361	0.9977	−0.1208	0.031*	
C10	0.84297 (16)	0.8279 (2)	−0.01997 (12)	0.0260 (4)	
H10A	0.9295	0.8221	0.0249	0.031*	
H10B	0.8302	0.7338	−0.0651	0.031*	
C11	0.74939 (15)	0.8130 (2)	0.03604 (12)	0.0237 (3)	
H11A	0.7669	0.7082	0.0732	0.028*	
H11B	0.6638	0.8048	−0.0102	0.028*	
C12	0.75418 (12)	0.9628 (2)	0.10585 (9)	0.0172 (3)	
C13	0.63162 (13)	0.9550 (2)	0.13481 (10)	0.0162 (3)	
H13A	0.5598	0.9545	0.0738	0.019*	
C14	0.62106 (15)	0.7897 (2)	0.18685 (12)	0.0222 (3)	
H14A	0.6961	0.7778	0.2440	0.027*	
H14B	0.6196	0.6944	0.1443	0.027*	
C15	0.50242 (14)	0.7810 (2)	0.21758 (11)	0.0193 (3)	
H15A	0.4268	0.7787	0.1603	0.023*	
H15B	0.5044	0.6770	0.2538	0.023*	
C16	0.49628 (13)	0.9347 (2)	0.28049 (9)	0.0163 (3)	
C17	0.36904 (13)	0.9597 (2)	0.30170 (10)	0.0177 (3)	
H17A	0.2992	0.9359	0.2414	0.021*	
C18	0.34553 (14)	0.8525 (2)	0.38237 (10)	0.0183 (3)	
H18A	0.4098	0.8847	0.4441	0.022*	
C19	0.21287 (14)	0.8933 (2)	0.38945 (10)	0.0210 (3)	
H19A	0.1925	1.0116	0.3720	0.025*	
H19B	0.1494	0.8235	0.3431	0.025*	
C20	0.20630 (14)	0.8619 (2)	0.49086 (10)	0.0199 (3)	
H20A	0.2328	0.7460	0.5101	0.024*	
H20B	0.2657	0.9377	0.5364	0.024*	
C21	0.07337 (13)	0.8897 (2)	0.49688 (10)	0.0185 (3)	
H21A	0.0137	0.8180	0.4489	0.022*	
H21B	0.0488	1.0071	0.4800	0.022*	
C22	0.06123 (14)	0.8520 (2)	0.59630 (10)	0.0181 (3)	
H22A	0.0879	0.7340	0.6134	0.022*	
C23	0.14585 (14)	0.9672 (2)	0.67541 (10)	0.0251 (3)	
H23A	0.1329	0.9426	0.7357	0.038*	
H23B	0.1247	1.0840	0.6582	0.038*	
H23C	0.2334	0.9476	0.6822	0.038*	
C24	−0.07746 (15)	0.8691 (2)	0.59158 (11)	0.0256 (3)	
H24A	−0.0841	0.8416	0.6536	0.038*	
H24B	−0.1296	0.7927	0.5434	0.038*	
H24C	−0.1057	0.9840	0.5747	0.038*	
C25	0.87768 (13)	0.9510 (2)	0.19492 (10)	0.0251 (3)	
H25A	0.9503	0.9631	0.1741	0.038*	
H25B	0.8810	0.8426	0.2259	0.038*	
H25C	0.8782	1.0402	0.2399	0.038*	
C26	0.60855 (13)	0.9319 (2)	0.37695 (10)	0.0218 (3)	
H26A	0.6867	0.9508	0.3644	0.033*	
H26B	0.6117	0.8232	0.4077	0.033*	
H26C	0.5971	1.0199	0.4188	0.033*	
C27	0.35678 (15)	0.6596 (2)	0.36912 (11)	0.0228 (3)	
H27A	0.4443	0.6303	0.3812	0.034*	
H27B	0.3073	0.6283	0.3040	0.034*	
H27C	0.3257	0.5997	0.4139	0.034*	

### Atomic displacement parameters (Å^2^)

**Table:**

	*U*^11^	*U*^22^	*U*^33^	*U*^12^	*U*^13^	*U*^23^
Cl1	0.0374 (2)	0.0516 (3)	0.02863 (18)	−0.0003 (2)	0.02143 (16)	−0.0012 (2)
O1	0.0309 (6)	0.0136 (5)	0.0292 (5)	0.0011 (5)	0.0125 (5)	0.0011 (4)
C1	0.0291 (8)	0.0188 (8)	0.0309 (8)	0.0032 (7)	0.0179 (6)	0.0008 (6)
C2	0.0282 (8)	0.0149 (8)	0.0264 (7)	0.0016 (6)	0.0137 (6)	−0.0009 (6)
C3	0.0173 (7)	0.0147 (7)	0.0171 (6)	0.0016 (6)	0.0059 (5)	0.0013 (5)
C4	0.0154 (6)	0.0123 (7)	0.0175 (6)	−0.0012 (5)	0.0047 (5)	−0.0011 (5)
C5	0.0199 (6)	0.0134 (7)	0.0180 (6)	−0.0039 (6)	0.0045 (5)	−0.0013 (5)
C6	0.0240 (7)	0.0178 (7)	0.0208 (6)	−0.0034 (6)	0.0086 (5)	0.0021 (6)
C7	0.0179 (7)	0.0202 (8)	0.0175 (6)	−0.0039 (6)	0.0059 (5)	−0.0011 (6)
C8	0.0240 (8)	0.0290 (10)	0.0251 (7)	−0.0031 (7)	0.0125 (6)	0.0017 (7)
C9	0.0214 (7)	0.0359 (10)	0.0230 (6)	−0.0014 (7)	0.0110 (5)	−0.0029 (7)
C10	0.0231 (8)	0.0296 (9)	0.0299 (8)	−0.0002 (7)	0.0150 (6)	−0.0062 (7)
C11	0.0230 (7)	0.0205 (8)	0.0317 (8)	−0.0021 (6)	0.0147 (6)	−0.0056 (6)
C12	0.0165 (6)	0.0169 (7)	0.0197 (6)	0.0001 (6)	0.0080 (5)	−0.0005 (6)
C13	0.0168 (6)	0.0123 (7)	0.0210 (6)	−0.0008 (5)	0.0084 (5)	−0.0014 (5)
C14	0.0274 (8)	0.0118 (7)	0.0341 (8)	0.0014 (6)	0.0192 (7)	0.0006 (6)
C15	0.0234 (7)	0.0121 (7)	0.0262 (7)	−0.0042 (6)	0.0133 (6)	−0.0019 (6)
C16	0.0158 (6)	0.0153 (7)	0.0178 (6)	0.0000 (6)	0.0058 (5)	0.0009 (5)
C17	0.0174 (6)	0.0174 (7)	0.0192 (6)	0.0017 (6)	0.0072 (5)	0.0016 (5)
C18	0.0180 (6)	0.0209 (8)	0.0176 (6)	0.0011 (6)	0.0079 (5)	0.0024 (5)
C19	0.0199 (7)	0.0250 (8)	0.0201 (6)	0.0023 (6)	0.0093 (5)	0.0028 (6)
C20	0.0183 (7)	0.0232 (8)	0.0195 (6)	0.0011 (6)	0.0079 (5)	0.0017 (6)
C21	0.0179 (6)	0.0191 (7)	0.0188 (6)	0.0007 (6)	0.0067 (5)	0.0018 (6)
C22	0.0221 (7)	0.0155 (7)	0.0182 (6)	−0.0008 (6)	0.0087 (5)	−0.0004 (5)
C23	0.0273 (7)	0.0244 (9)	0.0222 (6)	0.0014 (7)	0.0063 (5)	−0.0032 (6)
C24	0.0233 (7)	0.0289 (9)	0.0285 (7)	0.0000 (7)	0.0138 (6)	0.0015 (7)
C25	0.0182 (6)	0.0325 (9)	0.0239 (6)	0.0013 (7)	0.0064 (5)	0.0038 (7)
C26	0.0200 (6)	0.0222 (8)	0.0212 (6)	−0.0005 (6)	0.0045 (5)	0.0038 (6)
C27	0.0270 (7)	0.0189 (8)	0.0262 (7)	−0.0003 (7)	0.0136 (6)	0.0028 (6)

### Geometric parameters (Å, °)

**Table:**

Cl1—C9	1.8143 (14)	C15—C16	1.533 (2)
O1—C5	1.2203 (19)	C15—H15A	0.9700
C1—C2	1.549 (2)	C15—H15B	0.9700
C1—C17	1.562 (2)	C16—C26	1.5420 (19)
C1—H1A	0.9700	C16—C17	1.5627 (18)
C1—H1B	0.9700	C17—C18	1.5423 (19)
C2—C3	1.531 (2)	C17—H17A	0.9800
C2—H2A	0.9700	C18—C27	1.538 (2)
C2—H2B	0.9700	C18—C19	1.550 (2)
C3—C4	1.5354 (19)	C18—H18A	0.9800
C3—C16	1.548 (2)	C19—C20	1.5299 (19)
C3—H3A	0.9800	C19—H19A	0.9700
C4—C5	1.523 (2)	C19—H19B	0.9700
C4—C13	1.551 (2)	C20—C21	1.5295 (19)
C4—H4A	0.9800	C20—H20A	0.9700
C5—C6	1.4728 (19)	C20—H20B	0.9700
C6—C7	1.342 (2)	C21—C22	1.5342 (18)
C6—H6A	0.9300	C21—H21A	0.9700
C7—C8	1.518 (2)	C21—H21B	0.9700
C7—C12	1.519 (2)	C22—C23	1.523 (2)
C8—C9	1.521 (2)	C22—C24	1.530 (2)
C8—H8A	0.9700	C22—H22A	0.9800
C8—H8B	0.9700	C23—H23A	0.9600
C9—C10	1.510 (3)	C23—H23B	0.9600
C9—H9A	0.9800	C23—H23C	0.9600
C10—C11	1.532 (2)	C24—H24A	0.9600
C10—H10A	0.9700	C24—H24B	0.9600
C10—H10B	0.9700	C24—H24C	0.9600
C11—C12	1.548 (2)	C25—H25A	0.9600
C11—H11A	0.9700	C25—H25B	0.9600
C11—H11B	0.9700	C25—H25C	0.9600
C12—C25	1.5501 (19)	C26—H26A	0.9600
C12—C13	1.5614 (17)	C26—H26B	0.9600
C13—C14	1.530 (2)	C26—H26C	0.9600
C13—H13A	0.9800	C27—H27A	0.9600
C14—C15	1.5344 (19)	C27—H27B	0.9600
C14—H14A	0.9700	C27—H27C	0.9600
C14—H14B	0.9700		
			
C2—C1—C17	107.13 (12)	C14—C15—H15A	109.5
C2—C1—H1A	110.3	C16—C15—H15B	109.5
C17—C1—H1A	110.3	C14—C15—H15B	109.5
C2—C1—H1B	110.3	H15A—C15—H15B	108.0
C17—C1—H1B	110.3	C15—C16—C26	110.65 (13)
H1A—C1—H1B	108.5	C15—C16—C3	107.93 (10)
C3—C2—C1	103.42 (13)	C26—C16—C3	111.98 (12)
C3—C2—H2A	111.1	C15—C16—C17	116.36 (12)
C1—C2—H2A	111.1	C26—C16—C17	109.45 (11)
C3—C2—H2B	111.1	C3—C16—C17	100.07 (11)
C1—C2—H2B	111.1	C18—C17—C1	112.14 (12)
H2A—C2—H2B	109.0	C18—C17—C16	118.76 (12)
C2—C3—C4	119.15 (12)	C1—C17—C16	103.43 (11)
C2—C3—C16	103.82 (11)	C18—C17—H17A	107.3
C4—C3—C16	113.47 (11)	C1—C17—H17A	107.3
C2—C3—H3A	106.5	C16—C17—H17A	107.3
C4—C3—H3A	106.5	C27—C18—C17	113.57 (12)
C16—C3—H3A	106.5	C27—C18—C19	109.40 (13)
C5—C4—C3	112.97 (12)	C17—C18—C19	110.37 (12)
C5—C4—C13	108.62 (11)	C27—C18—H18A	107.8
C3—C4—C13	110.43 (11)	C17—C18—H18A	107.8
C5—C4—H4A	108.2	C19—C18—H18A	107.8
C3—C4—H4A	108.2	C20—C19—C18	112.80 (12)
C13—C4—H4A	108.2	C20—C19—H19A	109.0
O1—C5—C6	119.88 (14)	C18—C19—H19A	109.0
O1—C5—C4	123.22 (13)	C20—C19—H19B	109.0
C6—C5—C4	116.90 (13)	C18—C19—H19B	109.0
C7—C6—C5	123.69 (15)	H19A—C19—H19B	107.8
C7—C6—H6A	118.2	C21—C20—C19	113.23 (12)
C5—C6—H6A	118.2	C21—C20—H20A	108.9
C6—C7—C8	119.56 (14)	C19—C20—H20A	108.9
C6—C7—C12	123.02 (13)	C21—C20—H20B	108.9
C8—C7—C12	117.36 (13)	C19—C20—H20B	108.9
C7—C8—C9	111.24 (13)	H20A—C20—H20B	107.7
C7—C8—H8A	109.4	C20—C21—C22	114.93 (12)
C9—C8—H8A	109.4	C20—C21—H21A	108.5
C7—C8—H8B	109.4	C22—C21—H21A	108.5
C9—C8—H8B	109.4	C20—C21—H21B	108.5
H8A—C8—H8B	108.0	C22—C21—H21B	108.5
C10—C9—C8	110.69 (12)	H21A—C21—H21B	107.5
C10—C9—Cl1	109.97 (11)	C23—C22—C24	109.99 (12)
C8—C9—Cl1	109.26 (11)	C23—C22—C21	112.17 (12)
C10—C9—H9A	109.0	C24—C22—C21	110.39 (12)
C8—C9—H9A	109.0	C23—C22—H22A	108.1
Cl1—C9—H9A	109.0	C24—C22—H22A	108.1
C9—C10—C11	110.22 (13)	C21—C22—H22A	108.1
C9—C10—H10A	109.6	C22—C23—H23A	109.5
C11—C10—H10A	109.6	C22—C23—H23B	109.5
C9—C10—H10B	109.6	H23A—C23—H23B	109.5
C11—C10—H10B	109.6	C22—C23—H23C	109.5
H10A—C10—H10B	108.1	H23A—C23—H23C	109.5
C10—C11—C12	114.56 (13)	H23B—C23—H23C	109.5
C10—C11—H11A	108.6	C22—C24—H24A	109.5
C12—C11—H11A	108.6	C22—C24—H24B	109.5
C10—C11—H11B	108.6	H24A—C24—H24B	109.5
C12—C11—H11B	108.6	C22—C24—H24C	109.5
H11A—C11—H11B	107.6	H24A—C24—H24C	109.5
C7—C12—C11	110.19 (11)	H24B—C24—H24C	109.5
C7—C12—C25	107.75 (12)	C12—C25—H25A	109.5
C11—C12—C25	109.54 (13)	C12—C25—H25B	109.5
C7—C12—C13	108.94 (12)	H25A—C25—H25B	109.5
C11—C12—C13	107.99 (12)	C12—C25—H25C	109.5
C25—C12—C13	112.42 (11)	H25A—C25—H25C	109.5
C14—C13—C4	112.72 (11)	H25B—C25—H25C	109.5
C14—C13—C12	112.87 (12)	C16—C26—H26A	109.5
C4—C13—C12	112.63 (12)	C16—C26—H26B	109.5
C14—C13—H13A	106.0	H26A—C26—H26B	109.5
C4—C13—H13A	106.0	C16—C26—H26C	109.5
C12—C13—H13A	106.0	H26A—C26—H26C	109.5
C13—C14—C15	113.63 (13)	H26B—C26—H26C	109.5
C13—C14—H14A	108.8	C18—C27—H27A	109.5
C15—C14—H14A	108.8	C18—C27—H27B	109.5
C13—C14—H14B	108.8	H27A—C27—H27B	109.5
C15—C14—H14B	108.8	C18—C27—H27C	109.5
H14A—C14—H14B	107.7	H27A—C27—H27C	109.5
C16—C15—C14	110.91 (12)	H27B—C27—H27C	109.5
C16—C15—H15A	109.5		
			
C17—C1—C2—C3	11.85 (16)	C11—C12—C13—C14	60.73 (15)
C1—C2—C3—C4	−164.30 (12)	C25—C12—C13—C14	−60.24 (17)
C1—C2—C3—C16	−36.94 (15)	C7—C12—C13—C4	−50.55 (14)
C2—C3—C4—C5	−60.75 (17)	C11—C12—C13—C4	−170.22 (12)
C16—C3—C4—C5	176.54 (11)	C25—C12—C13—C4	68.81 (17)
C2—C3—C4—C13	177.39 (12)	C4—C13—C14—C15	49.92 (16)
C16—C3—C4—C13	54.68 (15)	C12—C13—C14—C15	178.93 (12)
C3—C4—C5—O1	21.5 (2)	C13—C14—C15—C16	−55.38 (17)
C13—C4—C5—O1	144.42 (14)	C14—C15—C16—C26	−64.78 (16)
C3—C4—C5—C6	−158.47 (12)	C14—C15—C16—C3	58.06 (15)
C13—C4—C5—C6	−35.59 (16)	C14—C15—C16—C17	169.50 (12)
O1—C5—C6—C7	−172.50 (15)	C2—C3—C16—C15	169.53 (12)
C4—C5—C6—C7	7.5 (2)	C4—C3—C16—C15	−59.66 (15)
C5—C6—C7—C8	−177.02 (13)	C2—C3—C16—C26	−68.45 (14)
C5—C6—C7—C12	0.2 (2)	C4—C3—C16—C26	62.37 (15)
C6—C7—C8—C9	−133.34 (16)	C2—C3—C16—C17	47.42 (13)
C12—C7—C8—C9	49.29 (18)	C4—C3—C16—C17	178.24 (11)
C7—C8—C9—C10	−55.93 (17)	C2—C1—C17—C18	146.24 (12)
C7—C8—C9—Cl1	−177.18 (11)	C2—C1—C17—C16	17.11 (15)
C8—C9—C10—C11	59.34 (16)	C15—C16—C17—C18	80.39 (16)
Cl1—C9—C10—C11	−179.83 (11)	C26—C16—C17—C18	−45.94 (18)
C9—C10—C11—C12	−55.40 (18)	C3—C16—C17—C18	−163.70 (13)
C6—C7—C12—C11	139.49 (15)	C15—C16—C17—C1	−154.66 (13)
C8—C7—C12—C11	−43.24 (17)	C26—C16—C17—C1	79.02 (14)
C6—C7—C12—C25	−101.04 (16)	C3—C16—C17—C1	−38.74 (13)
C8—C7—C12—C25	76.23 (15)	C1—C17—C18—C27	−175.77 (13)
C6—C7—C12—C13	21.18 (19)	C16—C17—C18—C27	−55.15 (18)
C8—C7—C12—C13	−161.54 (12)	C1—C17—C18—C19	60.95 (16)
C10—C11—C12—C7	45.83 (17)	C16—C17—C18—C19	−178.43 (13)
C10—C11—C12—C25	−72.54 (17)	C27—C18—C19—C20	81.20 (16)
C10—C11—C12—C13	164.71 (13)	C17—C18—C19—C20	−153.13 (14)
C5—C4—C13—C14	−172.73 (12)	C18—C19—C20—C21	−175.93 (14)
C3—C4—C13—C14	−48.33 (15)	C19—C20—C21—C22	177.44 (14)
C5—C4—C13—C12	58.15 (15)	C20—C21—C22—C23	61.84 (18)
C3—C4—C13—C12	−177.45 (11)	C20—C21—C22—C24	−175.12 (13)
C7—C12—C13—C14	−179.60 (12)		

### Hydrogen-bond geometry (Å, °)

**Table:**

*D*—H···*A*	*D*—H	H···*A*	*D*···*A*	*D*—H···*A*
C11—H11B···O1^i^	0.97	2.45	3.377 (2)	159
C14—H14B···O1^ii^	0.97	2.49	3.268 (2)	137

Symmetry codes: (i) −*x*+1, *y*−1/2, −*z*; (ii) *x*, *y*−1, *z*.
